# Pennsylvania State Dental Society

**Published:** 1888-08

**Authors:** 


Subject passed.

(TO BE CONTINUED.)

---

### PENNSYLVANIA STATE DENTAL SOCIETY.

### TWENTIETH ANNUAL MEETING HELD IN PHILADELPHIA, JUNE 5, 6, 7, 8, 1888.

ESPECIALLY REPORTED FOR THE INDEPENDENT PRACTITIONER.

### WEDNESDAY AFTERNOON SESSION.

DISCUSSION ON DR. SUDDUTH'S PAPER, ENTITLED "THE TREND OF DENTAL THERAPEUSIS."

*Dr. G. S. Allan*, of New York—There is no question, whatever, but that the dental profession is fully abreast of the times in all that pertains to therapeutics and materia medica. We make use of everything offered in the way of antiseptics or germicides, and we have among our members men of the greatest ability, whose researches have enriched, not only our own literature, but that of medicine also. The science of bacteriology seems to be all-pervading. One can hardly take up a paper or talk with a stranger without being amazed at the wide-spread influence of "bugs," as some roughly call them—bugs in the air, bugs in the water we drink, bugs everywhere.

We are indebted to Dr. Miller for what we know in regard to the etiology of caries, and also for the basis upon which to establish a line of preventive treatment, and yet he has not told us all, for he, himself, says that our knowledge is only in its infancy. Nevertheless, upwards of sixty separate forms of micro-organisms found in the mouth have been isolated and studied. It is true that many of these are harmless in character, a few give off lactic acid as a waste product, and thus produce decay, as a result of their growth. No other theory has ever been promulgated that will account for the formation of cavities other than the one given us by Dr. Miller.

Dr. Sudduth has truly said that the trend of our practice should be in the direction of prevention. I make use of antiseptics in my practice constantly, which I find especially applicable in extraction. Dr. Miller has called attention to its application here. You all, no doubt, have had unpleasant experiences—inflammatory results—tedious cures. Much trouble may be avoided by the use of antiseptic mouth washes and gargles, which must be used before and after extraction.

In opening into pulps my first act is to sterilize the cavity with a $\frac{1}{5}$ of 1 per cent. sol. bichloride of mercury, and then take every care not to force any of the contents of the pulp chamber through the apical foramen. I invariably dip my barbed points in the bichloride solution, as also all instruments used in removing dead pulps. I use the same precaution in the treatment of exposed pulps. I believe that fully one-half of the cases of failure after exposure is due to infection at the time of exposure, if the case be one of instrumentation. The moment I expose a pulp I touch it with bichloride solution, and afterwards seal it up with ether and rosin as a protective coating.

In the treatment of pyorrhœa alveolaris, antiseptic treatment comes in very happily. My first object is to remove the accumulations of tartar as well as possible with "cold steel," and then pass up around the root a sharp pointed stick saturated with a solution of 1 part pure sulphuric acid to 35 parts water. This is sufficiently strong to dissolve the tartar, but does not injure the gums or soft tissues.

*Dr. Magill,* Erie, Pa.—Dr. Allan's remarks were of great interest to me. I have been using hydronaphthol for some time with great satisfaction. I also use acids in the treatment of pyorrhœa alveolaris and think that good results may be obtained from the use of bichloride of mercury.

*Dr. Faught,* Philadelphia—I have used the antiseptic tablets, introduced by Dr. Carl Seiler, and find that they make a very agreeable mouth wash, and one that can be recommended.

*Dr. Kirk,* Philadelphia—I desire to call attention to the use of chloride of zinc in the treatment of pyorrhœa alveolaris. My attention was first directed to it by Dr. Essig. I have had a very satisfactory experience with it in a number of bad cases, using it in the strength of from 2½ to 3 grains to the ounce. One patient, a lady, had it in daily use for nearly a year. When she first came to me I thought that she would have to lose the lower incisors. After removing the tartar I dismissed her, with the prescription as above given. At the end of the time the gums had reattached themselves to the teeth. There had been no "creeping up" of the membrane; the teeth were simply tightened and answered all the requirements of mastication. The improvement in the condition of the mucous membrane was remarkable.

*Dr. James Truman,* Philadelphia—This is a subject that perhaps interests me more than any other. I do not think that the dental profession has paid the attention to it in the past that it deserves, and it must have larger observation in the future. We are liable, however, to make mistakes in our conclusions. I noticed one remark made by Dr. Sudduth, with which I cannot agree, viz.: "That the mouth is not the place for micro-organisms." I believe that micro-organisms have an important function to play in digestion and other matters connected with the oral cavity. It seems to me that we are running to extremes in the matter. It has been well said that their waste products will eventually destroy them, and some kinds may destroy others. In regard to antiseptics it is possible to go to too great extremes. It is important that the mouth be kept in a healthy condition, and an antiseptic used once in the twenty-four hours. The great fault I find with dentists generally is that they are "routinists;" they begin with some one agent, it may be creosote or iodoform, or some other, and they continue with it year in and year out. There have been a great many antiseptics introduced within the last three years—some inhibitory—others germicidal in character.

I do not believe in confining oneself to an agent so poisonous as bichloride of mercury. I know of no better agent to use than hydronaphthol. It is perfectly harmless. I prescribe it as a mouth wash and for cleansing rubber plates. Dr. Black first pointed out that the inflammation in mouths where rubber plates were worn was due to micro-organisms, and I can fully substantiate his observations. In cases where I have used hydronaphthol the mouth was free from inflammation. I believe in simple formulas, and do not use complex prescriptions. I believe that tooth powders, no matter whether antiseptic or not, are injurious. An irritation can not be kept up continuously without finally resulting in gingivitis. I know that in holding these views I oppose many, perhaps all. I do not interdict their use because they injure the enamel, but because of the irritation to the gums that may subsequently result in pyorrhœa. I use sulphuric acid—not the aromatic, but the commercial form, in the treatment of pyorrhœa. I do not allow it to remain long upon the gums, but follow it up with bicarbonate of soda, which, by its effervescence, throws out the debris better than peroxide of hydrogen. Pyorrhœa alveolaris is caused by micro-organisms, and must be treated in an entirely different manner from serumal deposits.

*Dr. Allan*—I confess I dissent very positively from what Dr. Truman has said. I do not see how a tooth can be kept clean without tooth powders, and after many years of personal use I fail to find any deleterious results from their use. I desire to state positively my belief that pyorrhœa alveolaris is always preceded by a deposit of serumal tartar, without which it does not occur. The first thing to do in treatment of any disease is to remove the cause. I do not believe that the most careful manipulation with steel instruments will accomplish this in all cases. Therefore I use sulphuric acid as my sheet anchor.

*Dr. Sudduth*—The deposits of the salts of calcium in pyorrhœa alveolaris are, in my opinion, the sequence and not the cause of the disease. I have failed to find the first case of serumic deposits where there was not evidence of previous catarrhal inflammation. Wherever chronic inflammation occurs, there lime salts are apt to be deposited. This often occurs in the eye and the brain. I

have never found serumic deposits beneath a perfectly healthy gum.

*Dr. Peirce*—I believe with Dr. Sudduth, that pyorrhœa alveolaris is a systemic condition, and that the tendency thereto is often inherited. I believe that the lime salts are a sequence and not the cause of pyorrhœa, and the micro-organisms are an accompaniment of inflammatory conditions, because they find in such conditions a suitable pabulum for their growth. They do not take hold of healthy tissues, but are found in diseased tissues, and exaggerate the already depraved condition by their presence. If we can keep the parts antisepticised we may modify the disease, but so long as the general systemic condition exists there is apt to be a recurrence. I do not know of any better method of treatment than that mentioned by Dr. Allan. I also use chloride of zinc, and am very much in favor of it because of its astringent and antiseptic action. I sometimes use it in the treatment of abscesses in the strength of from 3 to 4 grains to the ounce.

*Dr. Guilford,* Philadelphia—In regard to the treatment of exposed pulps a great many methods have been advocated. Some years since Dr. King, of Pittsburg, used oxide of zinc, made into a paste with carbolic acid. Many use it to-day and speak very favorably of it. When a pulp is exposed we have to be careful to leave no space for it to strangle itself in, also to make no pressure upon the pulp, because in so doing we only aggravate the inflammation and cause the death of the pulp. Always try to prevent subsequent irritation. The best method of treating exposed pulps was introduced by Dr. C. E. Francis, of New York, who uses paper disks saturated with canada balsam, dissolved in chloroform. The balsam causes the capping to adhere to the walls of the cavity. I have modified this by using several thicknesses of bibulous paper, which can be more easily adapted to the cavity; another very good application consists of a paste made of sulphate of morphia crystals, mixed with a sufficient quantity of carbolic acid to form a paste. The carbolic acid no doubt acts as a germicide, although I had not thought of it in that light. At any rate I have been exceedingly well satisfied with the results of treatment. While I have had some failures, yet the general results have been good.

*Dr. Peirce*—I use carbolic acid diluted with a small quantity of chloride of zinc, which coagulates a layer of albumen upon the surface and forms a good non-conducting capping that is not readily soluble and which does not make a good culture media for micro-organism; besides, the solution serves as a germicide.

*Dr. Faught*—I have had some experience in the treatment of pulps after amputating a portion. I use a small piece of bibulous paper saturated with oil of eucalyptus, which acts as a sedative, and is non-irritating. One case where I amputated a portion of the pulp in a superior molar has been under observation for more than a year, and I have every reason to believe it is in good condition.

*Dr. Ward,* Philadelphia—I have used disks made from printing paper as a capping for exposed pulp, with good results.

*Dr. C. B. Kratzer*—Following the practice of Dr. Smith, of Pittsburg, I, with several others, have used paper disks, saturated with pure wood creosote, not carbolic acid, for several years with marked success.

Subject passed.

MORNING SESSION, JUNE 6, 1888.

Discussion on Dr. Truman's paper (printed elsewhere in present number).

*Dr. Peirce*—The subject under consideration is one of great interest. I object to the proposition that cohesive foil is the best foil for students to begin with, because they have to use the mallet, and consequently do not learn that delicate touch that is required in adapting the gold to the walls of the cavity, which is acquired by hand pressure and soft foil. The method I pursue and teach my students is to first line the cavity with soft foil and work it into position by hand pressure. Then to finish with cohesive pellets. I think by this method better results may be obtained than by any other. Another objection to cohesive foil, in the hands of students, is found in the liability of the gold to ball because of its hardness. If fillings are taken out it will be discovered that this has occurred around the edges where little inequalities reveal that the gold has bridged in places.

*Dr. Guilford*—Dr. Truman uses the term soft foil in a way that I would not. Dr. Peirce also used the term cohesive differently from what I do. Confusion is liable to occur from the loose use of terms. In the first place, by cohesive foil we mean a pure gold foil, the particles of which will unite even at the slightest touch; on the other hand, non-cohesive foil should be included under that class of foils where the different particles do not adhere. As marked examples of such foils two may be mentioned as being on the market. Williams, of New York, cohesive, and Abbey, of Philadelphia, non-cohesive or soft. When this latter foil is folded it does not cohere, even if pressure is brought to bear upon it. It is absolutely non-cohesive. Intermediate between these two is another kind, that mentioned by Dr. Truman this morning, and which was in use when I entered the profession some twenty-five years since—a gold with very soft working qualities, but which when pressure is used is slightly cohesive, sufficiently so to hold it in place. It will not ball up or curl under the instrument. It spreads nicely under pressure, giving great adaptability. Soft foil cannot be used except in simple cavities having good walls. Strictly cohesive foil is objectionable because of its non-adaptability. We get the good qualities of each in semi-cohesive foil. It has the adaptability of non-cohesive foil and sufficient cohesive qualities to keep its position. When greater cohesiveness is required it may be obtained by heating it. If strictly non-cohesive foil is used to line cavities it is apt to be displaced—whereas semi-cohesive foil will remain where you put it.

Dr. Peirce advocates using non-cohesive foil around the margins of the cavities; this involves difficulties that may be overcome by the use of semi-cohesive foil, with which the same results may be obtained. Others have resorted to the use of tin for the cervical margins of cavities, filling over it with gold. I do not find this necessary. I can get just as good results with semi-cohesive foil. In regard to teaching students, I think their instruction should be as broad as possible, yet one particular method which is considered best should be dwelt upon, because the time of attendance upon instruction and also the capacity of students to grasp ideas is limited. I believe that one particular method should be emphasized, not, however, ignoring others. The fullest clinical instruction should be afforded by different operators.

*Dr. Magill*—The essayist alluded to influences that point toward a reaction in the profession to-day. I have observed the same myself. In looking over the past experience of the profession we see a most wonderful development. The cohesive qualities of foil gave a great impetus to contour work. The enthusiasm of operators and the prosperous times following the war combined to accomplish results that would not be submitted to at the present day. The reaction has come, both upon the part of the profession and the public, calling for lesser operations. It is a conservative movement toward a more sensible basis. I think the position Dr. Guilford takes is wrong. Dr. Truman is right. Dr. Truman says it is non-cohesive or cohesive foil, and the methods which are successful with non-cohesive foil are different from those which meet with success in the use of cohesive foil. I recognize them as two different systems. I do not mix them. It is cohesive or non-cohesive. If I build upon a non-cohesive filling it is a compromise, but if I take the course that Dr. Guilford pursues, it is nothing but a cohesive filling. Now in the use of cohesive foil we recognize from the start that there is nothing like that. It must be perfect from first to last. In building up the cohesive filling we build upon cohesive principles. I admit there are advantages in the use of semi-cohesive foil, but when you take issue with Dr. Peirce, where he uses a non-cohesive partly and a cohesive partly, I do not see where we are bettered by having his method than by using semi-cohesive. I have come to the conclusion that as a tooth saver non-cohesive is better than cohesive. I put it this way: The dentist does better work with non-cohesive foil than cohesive, but cohesive is the acme if you can get a man with the hand to make it perfect work.

*Dr. J. S. Smith*—Theories are all right if we could only put them into practice. That is what is wanted. We want men who will take the subject and make it practical. I find that the soft foil spoken of is a good thing for lining cavities. I believe that the cohesive foil used in lining the margins and at certain points at the cervical wall will produce failures, no matter how careful the manipulator may be. It depends largely upon manipulative skill in all cases of filling. My method has always been to use soft gold in retaining pits and to line walls; and as for finishing, I can do that with cohesive or semi-cohesive. It does not matter whether we have the two grades of foil or not. I would sooner have two grades right than one grade wrong. I have been using what is called a soft cohesive foil. I find it is a good foil to use, and it has been doing well in my hands. I do not believe in this thing of changing manufacturers of gold foil. I do not wish to be misunderstood in this respect, but I have adhered to one make for at least fourteen years. In the matter of filling teeth with cohesive foil we must commence right, and must carry the operations entirely through. As far as that is concerned, there is an incentive in teaching students to manipulate. They should learn how to use the mallet, and I always believe in the electric mallet. I think a young man should take a year's training with the hand mallet to obtain the sense of touch. I believe he will become a better manipulator.

*Dr. Gerhart*—The remarks that were made by Dr. Magill were eminently just. It must not be forgotten that the final object of filling is not ornamentation but conservation—that is, the arrestation of decay, and when we look to the value of different varieties of gold, it is necessary to take a broader view than simply isolated cases. When you find a community where operators have confined themselves to one kind of gold and another community where other kinds of gold have been used, one of the safest ways to determine the different value of these two kinds of gold is to note the differences in the proportion of artificial teeth worn in these communities. I know a community where two operators controlled a great deal of work for a number of years, and where soft foil has been used almost exclusively. I can say conclusively that in that community there are fewer people between fifteen and forty years of age wearing artificial dentures than in any other community I have ever seen. I think this is a point of value.

*Dr. Kingsbury*—I commenced the practice of our profession at eighteen years of age, and have filled teeth almost continuously from that time to the present, and having been an industrious worker, I presume have had as much experience in filling teeth with non-cohesive gold as any one in the room, and yet I would not undertake to say that my opinion should overrate that of others by any means.

If there is any virtue in using one kind for a long period of time I have enjoyed that privilege, for I have used Abbey's foil for a long time. When I first commenced the use of that foil it was the one gold foil manufactured in the world. There were very few manufacturers of gold foil in 1838—very few—and this foil went all over the world—China, mostly Japan—for there were a few dentists there, and to Europe—to France, although in Germany and France they filled teeth largely with lead, and in other kingdoms of Europe they used amalgam principally, for it had come into use before that time. Great changes have come since that first gold filling. Different varieties of gold have have come up. There are many cases where cohesive foil is indicated and should be used, but there are a larger number of cases by far where non-cohesive or soft gold is indicated and will do better service. I think it is an established fact that the better the filling is adapted to the walls of the cavity, and the more effectually the cavity is sealed against moisture and air, the more perfectly we preserve the tooth, other conditions being equal, and on account of the superior mobility of soft or non-cohesive gold over the cohesive. I think it is a much easier task to make a more perfect filling with non-cohesive foil in a large proportion of cases than with cohesive foil. I think a very great mistake is made on the part of the profession in relying upon certain points or cavities made at different distances, commencing with cohesive gold and filling the cavities in that way. I think the better plan is always to make the usual form of the cavity favorable for the retention of the filling, and not be dependent upon these little anchorage points. Make the general form of the cavity of the tooth such as will be maintained by the wedging process of the filling you insert. Such teeth will be better preserved. Probably I have not had as much experience in the use of cohesive foil and building up the crowns of teeth as some of those present.

I have built up a number of crowns with gold. We have a great many patients that will not endure these operations, nor are they willing to remunerate us for the length of time and skill required. I am greatly in favor of gold foil in all those cases where the general form of the cavity will maintain the filling without the aid of pits, so much used by many operators. Many teeth have been filled with cohesive foil and the fillings come out, leaving a little gold in the anchorage. This is a strong argument for the use of soft foil, depending, as it does, upon the shape of the cavity for its retention more than upon retaining points. In forming these cavities operators often injure the pulp and cause devitalization. I feel that more teeth are preserved by being filled with non-cohesive than by the use of cohesive gold foil. I do not stand here as a champion for gold over all other fillings. I think teeth may be saved by plastic fillings, and other metallic fillings as well as gold, but gold is the ne plus ultra of all material we use, and I am very partial to its use.

